# Investigating the advantages and disadvantages of electronic logbooks for education goals promotion in medical sciences students: A systematic review

**DOI:** 10.1002/hsr2.1776

**Published:** 2023-12-19

**Authors:** Somayeh Paydar, Erfan Esmaeeli, Fatemeh Ameri, Azam Sabahi, Marzieh Meraji

**Affiliations:** ^1^ Department of Health Information Technology, School of Allied Medical Sciences Kermanshah University of Medical Sciences Kermanshah Iran; ^2^ Health Information Management Department, School of Allied Medical Sciences Tehran University of Medical Sciences Tehran Iran; ^3^ Department of Health Information Technology, School of Paramedical Sciences, Student Research Committee Mashhad University of Medical Sciences Mashhad Iran; ^4^ Department of Health Information Technology, Ferdows Faculty of Medical Sciences Birjand University of Medical Sciences Birjand Iran; ^5^ Department of Health Information Technology and Medical Records, School of Paramedical Sciences Mashhad University of Medical Sciences Mashhad Iran

**Keywords:** E‐logbook, logbook, medical science students

## Abstract

**Background and Aims:**

Electronic logbook (E‐Logbook) is one of the practical software in medical science that serves as an auxiliary tool for comprehensive education, formative evaluation, and student learning documentation in clinical education. E‐logbooks are available to people on the Internet without any time or place restrictions. Experts' familiarity with e‐logbooks and their advantages and disadvantages can be effective in their better design so professors and students can use their potential benefits. Therefore, this study examines the advantages and disadvantages of an e‐logbook.

**Methods:**

This systematic review was conducted until June 13, 2022, by searching relevant keywords such as logbook, e‐logbook, and medical students in PubMed, Scopus, and Web of Science databases. Data were extracted using the data extraction form. The contents of the studies were analyzed based on the study's aim. The results of the analyses were presented in the form of descriptive statistics (tables and figures).

**Results:**

Out of 365 retrieved studies, 13 were selected to investigate the advantages and disadvantages of e‐logbooks. Most studies were conducted in Pakistan (*n* = 4) and focused on medical students with different specialties (*n* = 10). The advantages and disadvantages of e‐logbooks were classified into nine and four categories, respectively. Most advantages of e‐logbooks were related to monitoring and evaluating the performance of students and instructors (*n* = 11). Their most disadvantages were associated with hardware and software (*n* = 8).

**Conclusion:**

According to the results, e‐logbooks can improve clinical education, provide feedback to people, control the achievement of educational goals, and increase professor−student interaction. Hence, it is recommended to address their disadvantages and barriers to improve the quality of students' performance.

## 1 INTRODUCTION

The value of an ideal clinical education in professional and personal advancement and the development of students' skills is undeniable.^1^ Clinical education during the internship period allows students to transform theoretical knowledge into various psychological‐motor skills required for patient care; thus, all students should be able to perform the acquired skills competently at the end of their studies.^2^, ^3^ Logbook is one of the most powerful tools for educating and evaluating students, especially medical students and related fields.^4^ A logbook is a notebook that includes the minimums required for planned education and recorded practical learning. It is completed by the learner (mentioning the date, time, and method of doing the work) and is monitored and evaluated by the relevant supervisor.^5^ Using the logbook provides a structure and concentration in learning and evaluation in a real experimental environment.^6^ Logbooks are helpful in providing feedback to students or interns, tracking their performance, and assisting them in planning their activities. By shortening the gap between theory and practice, they can help professors and students achieve educational and clinical goals.^7^, ^8^

An ideal logbook should be applicable and acceptable to students and provide a quick and accurate collection of data related to educational objectives for timely analysis and feedback to students.^9^ Logbooks assist students in becoming acquainted with learning tasks and goals, directing their efforts toward learning assigned tasks, directing teachers' efforts toward teaching students, fostering educational interaction between educator and learner, and documenting students' practical activities^10^, ^11^ E‐learning has grown in educational programs in recent years. The evaluation of students' learning is considered a vital issue, especially in practical skills and the quality of e‐learning content.^12^ Electronic logbook (E‐logbook) is one of the applicable software in medical education. It is an auxiliary tool in comprehensive education, formative evaluation, and the assessment of students' learning documentation in clinical education.^13^ They are generally digital adaptations of traditional logbooks used to collect and monitor data.^14^ In the traditional method, the user manually records the data in a logbook and creates a written report. Whereas in the novel approach, with the e‐logbook available, users easily enter data into the system, which automatically provides reports at different intervals.^14^, ^15^ Moreover, the latter can provide more accurate computational results than the former, requiring users to calculate the data.^16^ As a result, e‐logbook does not have the problems of manual recording.^15^ Another characteristic of the e‐logbook is that users can easily access it anywhere at any time. This platform allows users to record all relevant data, such as dates, events, and activities, in the cloud space for future use or review.^17^

Tamblyn et al. designed a mobile app‐based e‐logbook for trainees to reduce the complications of central venous catheter insertion. Smartphones were available in hospitals, and many clinicians used mobile apps as a source of instant access to information for clinical decision‐making. Hence, one of the most significant advantages of adding an e‐logbook to the mobile phone was quick data entry to save physicians time and energy.^18^ Kumar et al. investigated the use of an e‐logbook for postgraduate psychiatry training. They introduced two e‐logbook models for psychiatry training used based on the needs and resources available in institutions or departments. The first model was an offline e‐logbook without an internet connection. Data were manually entered and saved on the computer using database management software, depending on the available resources. The second was an online or real‐time e‐logbook using a database program or personal digital assistant with an internet‐connected device. One advantage of the latter was accessibility by multiple devices anywhere and anytime, with data usually backed up automatically. The online logbook allowed learners to view their performance, peer evaluations, and supervisor feedback in real‐time. This model had better acceptability, transparency, and flexibility for upgrading with advanced technology.^19^ Gondal et al. investigated supervisors' perspectives on the role of the e‐logbook in the College of Physicians and Surgeons in Pakistan to monitor the training of postgraduate medical residents. Their results revealed that the e‐logbook system had been widely accepted by supervisors with a positive perception of its usefulness. In the mentioned study, the common reasons that prevented regular feedback included unfamiliarity with the e‐logbook interface, internet access issues, busy schedules, and some considering the e‐logbook use as a cumbersome task.^20^

E‐logbooks are available to people on the Internet without any time or place restrictions. Experts familiarity with the e‐logbook and their awareness of its advantages and disadvantages (to be used in the design and implementation of e‐logbooks) can lead to the production and publication of logbooks successfully, thus providing a better educational monitoring system for professors and students. To the best of our knowledge, no study has been conducted on the advantages and disadvantages of e‐logbooks and their features. Thus, this study aims to review published articles to identify the advantages and disadvantages of e‐logbooks in medical sciences for promoting education goals. This study could lead to designing educational logbooks comprehensively and monitoring and evaluating students’ learning purposefully, quickly, and accurately.

## 2 METHODS

### 2.1 Databases and search strategy

This study followed the PRISMA guidelines^21^ to investigate the advantages and disadvantages of e‐logbooks. Web of Science, PubMed, and Scopus databases were searched to retrieve English articles. The searches were conducted without time restrictions until June 13, 2022. Two researchers designed the search strategy by combining two keywords, “e‐logbook” and “medical science student” (Table 1).

**Table 1:** **Search strategies for different databases**.

Database	Search strategy
PubMed	((logbook*[Title/Abstract] OR “electronic logbook*”[Title/Abstract] OR “e logbook*”[Title/Abstract] OR “e‐logbook*”[Title/Abstract]) AND (“medical sciences students”[All fields] OR “students of medical sciences” [All fields] OR “medical school students”[All fields] OR “medical students”[All fields] OR “students of medical school”[All fields] OR “medical faculty students”[All fields] OR students, medical [Mesh terms]))
Web of Science	((ALL = (“medical sciences students”) OR ALL = (“students of medical sciences”) OR ALL = (“medical school students”) OR ALL = (“medical students”) OR ALL = (“students of medical school”) OR ALL = (“medical faculty students”)) AND (TS = (“logbook*”) OR TS = (“electronic logbook*”) OR TS = (“e logbook*”) OR TS = (“e‐logbook*”)))
Scopus	((ALL (“medical sciences students) OR ALL(“students of medical sciences”) OR ALL (“medical school students”) OR ALL (“medical students”) OR ALL (“students of medical school”) OR ALL (“medical faculty students”)) AND (TITLE‐ABS‐KEY (“ logbook*”) OR TITLE‐ABS‐KEY (“electronic logbook*”) OR TITLE‐ABS‐KEY (“e logbook*”) OR TITLE‐ABS‐KEY (“e‐logbook*”)))

### 2.2 Inclusion and exclusion criteria

The inclusion criteria of the study included original English‐language articles introducing e‐logbooks with a focus on their advantages and disadvantages in the field of medical sciences. All short papers, letters to editors, conference abstracts, review articles, and papers whose full texts were unavailable were excluded. Additionally, the studies with a focus on designing paper‐based logbooks or e‐logbooks in fields other than medical sciences were excluded because they were not in line with the study goals.

### 2.3 Selection

The retrieved studies were entered into EndNote Ver 20. First, duplicate articles were identified and removed using the relevant software. Then, the title and abstract of all studies were reviewed according to the inclusion criteria, and their full texts were read, if necessary. Two researchers performed the study selection process independently, and any disagreements were referred to a third researcher.

### 2.4 Data extraction and analysis

After selecting the studies based on the inclusion and exclusion criteria, data were collected using the data extraction form in Excel (Microsoft, 2019) based on the study's objectives. These data included the name of the first authors, the year of studies, the country of studies, the purpose of studies, methodologies, and advantages and disadvantages of e‐Logbook. The studies were selected independently by two researchers, and in case of any disagreement, it was referred to the third researcher. The research team analyzed the study content based on the study's objectives. After reviewing previous studies,^11^, ^16^ the advantages of e‐logbooks were categorized into 52 items and their disadvantages into 22 items. The research team then held a focus group meeting with 10 experts (with at least 5 years of work experience), including 4 nursing PhDs, 4 health information management PhDs, and 2 medical informatics PhDs. The experts rated the importance of each item on a 5‐point Likert scale from “*strongly disagree*” to “*strongly agree*.” The average importance of items related to the advantages and disadvantages of e‐logbooks was estimated, and then the categories were finalized. The results of the analyses were presented as descriptive statistics (tables and figures).

### 2.5 Quality assessment

Evaluation of the studies quality was carried out using MERSQI (Medical Education Research Study Quality Instrument) Study quality was assessed using the Medical Education Research Quality Instrument (MERSQI) for quantitative studies, which is a validated tool widely used in education research.^22^, ^23^ This quality assessment tool comprises six domains: “study design,” “sampling,” “type of data,” “validity of evaluation instrument,” “data analysis,” and “outcomes.” The minimum score within five of the domains is = 1, and the maximum score across all domains is = 3. Accordingly, MERSQI scores range from 5 to 18. This quality assessment tool comprises six domains: “study design,” “sampling,” “type of data,” “validity of evaluation instrument,” “data analysis,” and “outcomes.” The minimum score within five of the domains is = 1, and the maximum score across all domains is = 3. Accordingly, MERSQI scores range from 5 to 18. Two independent authors (S. P. and A. S.) assessed the quality of each study and inconsistency was resolved by involving a third author (M. M.).

All ethical considerations such as getting written consent, maintaining data confidentiality at all stages, the possibility of excluding at any stage, and optional participation in the study were observed.

## 3 RESULTS

**Figure 1:**
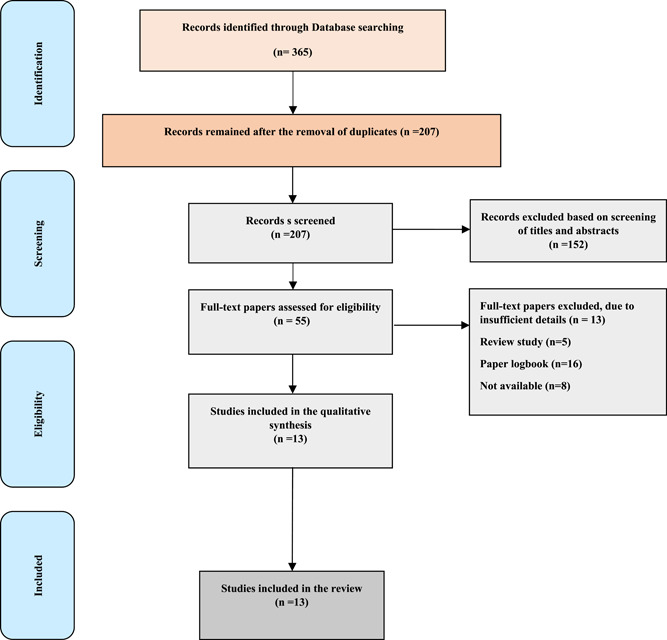
Flow diagram of the included and excluded studies.

**Figure 2:**
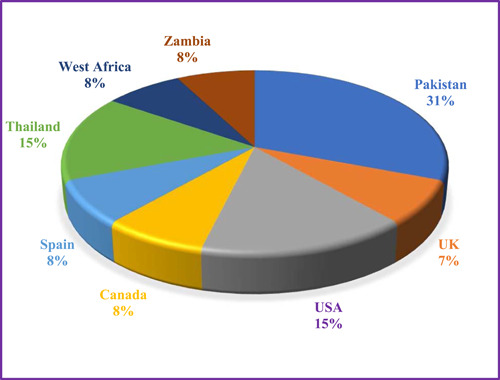
Frequency percentage of the studies conducted by countries.

A total of 365 articles were found in the initial search. After removing duplicates, unrelated studies were left out based on the evaluation of titles, abstracts, and full texts, and finally, 13 articles were selected to introduce or review the advantages and disadvantages of an e‐logbook. A summary of the search process and selection of the studies is presented in the PRISMA diagram (Figure 1). Figure 2 shows that most of the studies on e‐logbooks were conducted in Pakistan (31%).

Seventy‐six percent of the logbooks were designed for medical students with different specialties (8% for nursing students and 8% for anesthesia students). Moreover, in one study (8%), the e‐logbook was specific to medical and anesthesiology students (Table 2). Table 3 presents the advantages of using e‐logbooks. Based on this table, advantages were identified in 9 groups and 49 subgroups. The most advantages, with a frequency of 11, were related to supervising, monitoring, and evaluating the performance of the students and educators. Table 4 shows the disadvantages of e‐logbooks in 4 groups and 17 subgroups. The most disadvantages, with a frequency of 8, were related to hardware and software problems.

**Table 2:** Characteristics of the selected studies.

First author	Publication year	Country	Study objective	Field of study
Aphinives^24^	2013	Thailand	Implementing an e‐logbook to record trainees' general surgery experiences	Medical (general surgery specialty)
Viseskul^25^	2019	Thailand	Investigating the possibility of developing and allowing an experimental implementation of a logbook to monitor the progress of international PhD students	Nursing
Denton^26^	2007	USA	Determining the accuracy of listing students' problems in the logbook (measured based on sensitivity and specificity) and determining the completeness of patient entries by students	Medical
Gondal^8^	2020	Pakistan	Determining supervisors' feedback on the role of e‐logbook in monitoring training, strengthening supervisor−supervisor relationships, and improving the general setting of postgraduate education in the College of Physicians and Surgeons of Pakistan (CPSP)	Medical
McGinn^27^	2020	Canada	Evaluating an agreement between anesthesia information management systems (AIMS) and residents' logbooks (RLBs) for monitoring the provision of necessary care to patients during anesthesia residency	Anesthesia
Gray^28^	2019	UK	Evaluating how a logbook based on a smart mobile phone application helps reflection	Medical (obstetrics and gynecology)
Cottrell^29^	2010	USA	Investigating the number of student evaluators that is necessary for a reliable estimation of educators' approach to the clinical learning process in clinical settings	Medical
Díaz^30^	2015	Spain	Investigating the advantages and disadvantages of the surgical e‐logbook	Medical (surgery specialty)
Gondal^20^	2017	Pakistan	Investigating the supervisor's views on the role of the e‐logbook of the College of Physicians and Surgeons of Pakistan (CPSP) in supervising the education of CPSP postgraduate medical assistants	Medicine
Ullah^31^	2019	Pakistan	Investigating the supervisors' perceptions of the use of an e‐logbook (E‐Log) system used to monitor graduate education	Anesthesia, medicine (cardiac surgery, general surgery, maxillofacial surgery), obstetrics and gynecology, nephrology, ophthalmology, orthopedics, pathology, psychiatry, radiology, and urology
Iqbal^32^	2015	Pakistan	Recording the views of residents regarding the application of the new e‐logbook (E‐Log) system introduced in the College of Physicians and Surgeons of Pakistan (CPSP)	Medical
Barteit^11^	2022	Zambia	Evaluating the feasibility of this e‐logbook, its acceptability among a cohort of Medical Licentiate students and their mentors, as well as their facilitators and barriers	Medical
Sung^16^	2021	West Africa	Evaluating the quality of an app‐based surgical e‐logbook system shortly after its implementation in a low‐income country and identifying potential areas of improvement for the system	Medical (surgery)

Abbreviation: E‐Logbook, electronic logbook.

**Table 3:** **Advantages of e‐logbooks**.

Row	Main groups	Subgroups
1	Ease of data entry, storage, analysis, and various report generation	‐ Ease of searching words^24^ ‐ Analyzing various procedures among educational institutions^24^ ‐ Easy online recording of activities^25^, ^28^, ^30^ ‐ Fast data entry^16^, ^25^, ^30^ ‐ Ease of collecting, storing, and analyzing data^11^, ^16^, ^26^ ‐ Recording activities by courses, specialized units, levels of responsibility, and procedural complexity^30^ ‐ Generating immediate reports on various aspects of scientific and educational activities^24^, ^27^, ^30^ ‐ Providing reliable and accurate reports on educational activities^30^ ‐ Providing up‐to‐date, personal, or general reports automatically^30^
2	Ease of accessing, maintaining, and sharing information	‐ More efficient access to data^16^, ^24^, ^30^ ‐ Access to information at any time and place^11^, ^16^, ^25^ ‐ Indexing students' experiences^8^, ^29^ ‐ The ability to link data^28^ ‐ Ease of access to educational materials^8^, ^28^ ‐ Ease of maintaining e‐logbooks^31^
3	Supervising, monitoring, and evaluating students' and educators' performance	‐ Providing the conditions for continuous monitoring and evaluating students' performance^11^, ^25^, ^28^ ‐ Curriculum evaluation and development^26^ ‐ Monitoring student experiences during a clinical rotation^26^, ^27^ ‐ Comparison of student experiences in a multi‐department hospital internship unit^26^ ‐ Better performance monitoring than paper logbook^8^, ^20^ ‐ Better management of trainees' workflow^8^, ^11^ ‐ Getting an overview of student education^11^, ^30^ ‐ Supervising and monitoring education and educational program^31^, ^32^ ‐ Supervising and monitoring trainees and trainers^24^ ‐ Identifying educational gaps^32^
4	Providing feedback	‐ Trainees' awareness of the results of their examinations and providing timely feedback^20^, ^24^, ^28^, ^29^, ^32^ ‐ Examining students' answers to logbook questions^29^ ‐ Increasing students' efficiency by providing timely feedback^20^ ‐ Strengthening the curriculum and academic consultants to monitor and motivate students^25^, ^29^, ^32^
5	Improving the quality of educational processes	‐ Fast curriculum preparation by students^30^ ‐ Alignment of students with educational goals^26^ ‐ Organization of clinical activities required by the curriculum^20^ ‐ Professional growth^8^ ‐ Ease of memorization using recorded videos^28^ ‐ Improving clinical skills^20^ ‐ Improving educational standards^31^ ‐ Increasing concentration during the learning cycle^32^ ‐ Increasing students' decision‐making skills^8^
6	Improving communication and satisfaction	‐ Improving the relationship between supervisor and supervisee^8^ ‐ Creating a connection between students and educational items^29^ ‐ Increasing interaction with supervisors^32^ ‐ Increasing student satisfaction^8^, ^16^
7	Information control and security	‐ Preparing a backup copy^24^ ‐ Maintaining the student's anonymity in the logbook^29^
8	Reduction in costs	‐ Cost‐effective^8^, ^31^, ^32^ ‐ Reducing the financial costs of the organization^27^
9	Usability	‐ Flexibility^8^, ^31^ ‐ Easy user interface^32^ ‐ User‐friendly^11^, ^32^

Abbreviation: E‐Logbook, electronic logbook.

**Table 4:** **Disadvantages of e‐logbooks**.

Row	Main groups	subgroups
1	Hardware and software problems	‐ Dependence on the Internet^24^, ^31^, ^32^ ‐ Internet connection problems^16^, ^25^ ‐ Lack of enough space on the hard disk at the time of data entry^30^ ‐ Software barriers for data entry^11^, ^16^, ^26^
2	Documentation weakness, reduced information quality, and a lack of data quality control system	‐ The low accuracy of e‐logbooks in high‐risk evaluations^26^ ‐ Unreliability of recorded items in e‐logbooks^8^, ^27^, ^31^ ‐ Recording false and incorrect cases in e‐logbooks^27^ ‐ Reducing the reliability and validity of the logbook^31^ ‐ Failure to verify the accuracy of the patient's medical records^30^ ‐ Complete recording of surgeries instead of procedures^30^
3	Waste of time and resources	‐ Increasing the workload for data entry^25^ ‐ Spending more time accessing the information system^8^, ^25^, ^31^ ‐ Lack of time and resources to provide regular feedback through web‐based systems^11^, ^20^
4	Designing and learning problems	‐ Not suitable for people unfamiliar with electronic technologies^8^, ^16^ ‐ Not suitable for continuing student learning^29^ ‐ Designing for primary specializations and not paying attention to minor specializations^20^ ‐ The voluntary nature of the e‐logbook system^30^, ^31^

Abbreviation: E‐Logbook, electronic logbook.

## 4 DISCUSSION

Logbooks are verified files of learner progress documenting the acquisition of necessary knowledge, skills, attitudes, and competencies.^19^ They have two important uses: (1) they serve as a document of the training received by trainees, and (2) the data recorded in the logbook provides a large data set for monitoring and evaluating training.^33^ The present study investigates the advantages and disadvantages of e‐logbooks, presented in two groups and their subgroups.

### 4.1 Advantages of e‐logbooks

#### 4.1.1 Ease of data entry, storage, analysis, and various report generation

E‐logbooks provide the opportunity to enter data at the point of care for students. It also allows the end user to edit and customize headers, menus, and other datasets based on their needs, which is one of their desirable features.^34^ According to Watters et al., ideal e‐logbooks allow data entry and retrieval through various systems for trainees and automatically provide them with reports. These reports should cover the qualitative and educational aspects of their experience.^35^ In this study, online and easy recording of activities^25^, ^28^, ^30^ and real‐time report generation in various aspects of scientific and educational activities^24^, ^27^, ^30^ were also important advantages of this group mentioned in previous studies.

#### 4.1.2 Ease of accessing, maintaining, and sharing information

In this review, ease of accessing^11^, ^16^, ^24^, ^25^, ^30^ and maintaining information^31^ were among the e‐logbook advantages. Brouwer et al. revealed that the ease of recording information online resulted in educational progress and access to information at any time and place.^36^ Akhavan et al. also stated that an e‐logbook is recommended only by providing adequate equipment and training in information technology.^37^ Moreover, as regards sharing information in logbooks, Kumar et al. stated that this advantage provided a comprehensive report of student performance, along with other summative evaluations, to help educators make decisions^19^; this is consistent with the results of our study.

#### 4.1.3 Supervising, monitoring, and evaluating student and educator performance

Davarinia et al. showed that using an e‐logbook improved the evaluation of students' clinical performance in the internship environment by clinical professors. It also motivated students to enhance their clinical competence in performing procedures and their roles as anesthesiology and operating room students in apprenticeship settings, owing to the accurate and weekly assessment of the logbook checklists by professors.^38^ Their results were consistent with most of the selected articles in our review.

#### 4.1.4 Providing feedback

One of the significant characteristics of the e‐logbook is the opportunity to provide constructive feedback. It helps bridge the gap between current and desired understanding and leads to timely corrections and performance improvement in students.^39^ In the present study, the main advantages of the group were providing feedback, informing trainees of their examination results with timely feedback,^20^, ^24^, ^28^, ^29^, ^32^ and strengthening the curriculum and academic consultants to monitor and motivate students.^25^, ^29^, ^32^ Mohammadi also stated that the e‐logbook could help achieve educational and clinical goals and provide valuable feedback to students and professors.^7^

#### 4.1.5 Improving the quality of educational processes

Logbooks positively impact their teaching and learning process by providing a clear picture of what students gain during their learning experiences.^40^ In the present study, nine advantages were obtained in the group of improving the quality of educational processes. The results showed that e‐logbooks enhanced students' learning, concentration, skills, and professional growth. Some studies have reported that e‐logbooks promote students' teaching and learning structure, help their evaluation, and increase their professional growth and achievement if designed and used properly.^41^, ^42^, ^43^ Furthermore, the majority of anesthesiology and operating room students who participated in the study conducted by Davarinia et al. reported effective learning as one of the main benefits of the e‐logbook.^38^

#### 4.1.6 Improving communication and satisfaction

According to a study in which e‐logbooks were introduced to four centers in England by the Royal College of Psychiatrists, 80% of educators and 60% of students were satisfied with e‐logbooks owing to improving the training.^44^ Mazari et al. showed that 60% of dental students were satisfied with e‐logbooks, and cooperation and mutual communication between educators and students were necessary for this satisfaction.^45^ These results are in line with those of the present review.

#### 4.1.7 Information control and security

Eroes et al.^46^ and Kumar et al.^19^ mentioned in their studies that e‐logbooks facilitate making a backup copy and storing information on hard disks and in print form for storage in the archive. Furthermore, these types of logbooks have security features such as a username, a unique password (for each person), a one‐time password, and updated antivirus subscriptions since the security of e‐logbooks may be compromised due to third‐party access and data theft.^19^

In this review, preparing a backup copy of logbooks was one of the advantages of the information security and control group, which is in line with the results of the above two studies.

#### 4.1.8 Reduction in costs

An ideal logbook is cost‐effective, practical, and approved by students. It also allows for the quick and precise collection of data related to educational objectives to analyze the data and provide accurate feedback to students.^47^ Several studies introduced logbooks as a cost‐effective tool.^8^, ^31^, ^32^

#### 4.1.9 Usability

The user‐friendly^11^, ^32^ and easy user interface were among the advantages obtained in this review.^32^ Jameson et al.^48^ and Watters et al.^35^ also reported the e‐logbook as an online tool accepted by students due to its ease of use, convenience, and quick data entry

### 4.2 Disadvantages of e‐logbooks

#### 4.2.1 Hardware and software problems

Dependence on the Internet was one of the most significant software problems of e‐logbooks in this study.^24^, ^31^, ^32^ Kumar et al. classified the types of e‐logbooks into two groups: online and offline. Offline e‐logbooks do not need an internet connection and better data control and security. Their most significant disadvantage is the risk of data loss and the need for Internal and external hard disks to store data. In contrast, online logbooks use a database connected to the Internet, requiring expensive hardware and software tools to ensure data security.^19^

#### 4.2.2 Documentation weakness, reduced information quality, and lack of data quality control system

Our review showed that one of the primary disadvantages of logbooks in this group was the unreliability of the recorded items,^8^, ^27^, ^31^ which could be due to their structure. Svendsen et al. evaluated the quality of online surgical logbooks. According to their results, the collected information from surgical logbook systems should be used cautiously, and much effort should be made to provide reliable data by the used logbook systems.^49^ According to Denton et al., logbooks did not have the necessary reliability and validity to change educational programs and verify educational goals.^47^ Our study reported a decrease in the reliability and validity of logbooks as one of its disadvantages.

#### 4.2.3 Waste of time and resources

Svendsen et al. reported a large volume of data in logbooks as one reason for their unfriendliness; this problem can be solved by changing and improving the structure of e‐logbooks.^49^ While the present study found that e‐logbooks increased the workload for data entry, Davarinia et al. reported that the majority of anesthesiology (100%) and operating room (83%) students were satisfied with the time‐saving feature of e‐logbooks for recording their apprenticeship activities.^38^

#### 4.2.4 Designing and learning problems

The present review identified some issues with e‐logbook designing and learning, such as being unsuitable for continuing learning^29^ and designing for major rather than minor specialties.^20^ Regarding these problems, Busemann et al. also mentioned that although e‐logbooks are a powerful learning tool, they should be adapted to students' needs; otherwise, they may cause demotivation and learning lack.^50^

Despite the importance of this systematic review, one of the limitations of the current study was the few studies have been conducted on the advantages and disadvantages of e‐logbooks. Another limitation was that it only included articles written in English, and the inclusion of other language may have offered different results.

## 5 CONCLUSION

Based on the results, e‐logbooks, as a practical and useful evaluation tool, can promote education and improve educational processes and student learning. These logbooks increase the students' and educators' satisfaction owing to their user‐friendliness and access to information at any time and place. However, they have some disadvantages and problems. These disadvantages can be addressed by creating appropriate Internet access platforms and using technological tools to enter data to reduce the workload. This can be possible by improving the infrastructure (by each country's government because it is out of the possibility of a university). Moreover, the trainees' performance improves by enhancing the structure of logbooks, accurately and correctly recording the information, and receiving feedback.

## AUTHOR CONTRIBUTIONS

**Somayeh Paydar**: Conceptualization; data curation; formal analysis; writing—original draft; writing—review and editing. **Erfan Esmaeeli**: Data curation; formal analysis; methodology. **Fatemeh Ameri**: Data curation; formal analysis; methodology. **Azam Sabahi**: Conceptualization; methodology; supervision; validation; writing—original draft; writing—review and editing. **Marzieh Meraji**: Data curation; formal analysis.

## CONFLICT OF INTEREST STATEMENT

The authors declare no conflict of interest.

## ETHICS STATEMENT

All participants were informed about the pertinent study aspects and provided written informed consent. All methods were performed in accordance with the relevant guidelines and regulations.

## TRANSPARENCY STATEMENT

The lead author Azam Sabahi affirms that this manuscript is an honest, accurate, and transparent account of the study being reported; that no important aspects of the study have been omitted; and that any discrepancies from the study as planned (and, if relevant, registered) have been explained.

## Data Availability

All data generated or analyzed during this study are included in this published article. All authors have read and approved the final version of the manuscript had full access to all of the data in this study and takes complete responsibility for the integrity of the data and the accuracy of the data analysis.
